# Insights into the Neutrophil-to-Lymphocyte Ratio and the Platelet-to-Lymphocyte Ratio as Predictors for the Length of Stay and Readmission in Chronic Heart Failure Patients

**DOI:** 10.3390/diagnostics14182102

**Published:** 2024-09-23

**Authors:** Liviu Cristescu, Ioan Tilea, Dragos-Gabriel Iancu, Florin Stoica, Diana-Andreea Moldovan, Vincenzo Capriglione, Andreea Varga

**Affiliations:** 1 Faculty of Medicine in English, George Emil Palade University of Medicine, Pharmacy, Science and Technology of Targu Mures, 540142 Targu Mures, Romania; liviu.cristescu@umfst.ro (L.C.); diana.moldovan@umfst.ro (D.-A.M.); capriglione.vincenzo@stud18.umfst.ro (V.C.); andreea.varga@umfst.ro (A.V.); 2 Doctoral School, George Emil Palade University of Medicine, Pharmacy, Science and Technology of Targu Mures, 540142 Targu Mures, Romania; dragos-gabriel.iancu@umfst.ro (D.-G.I.); stoica.florin.23@stud.umfst.ro (F.S.); 3 Department of Internal Medicine II-Cardiology, Emergency Clinical County Hospital, 540042 Targu Mures, Romania; 4 Faculty of Medicine, George Emil Palade University of Medicine, Pharmacy, Science and Technology of Targu Mures, 540142 Targu Mures, Romania; 5 Department of Cardiology I, The Emergency Institute for Cardiovascular Diseases and Transplantation, 540136 Targu Mures, Romania

**Keywords:** neutrophil-to-lymphocyte ratio, platelet-to-lymphocyte ratio, N-terminal pro B-type natriuretic peptide, chronic heart failure, length of stay, readmission

## Abstract

**Background/Objectives:** Chronic heart failure (CHF) is characterized by complex pathophysiology, leading to increased hospitalizations and mortality. Inflammatory biomarkers such as the neutrophil-to-lymphocyte ratio (NLR) and platelet-to-lymphocyte ratio (PLR) provide valuable diagnostic insights. Methods: This study evaluates the prognostic relationship between NLR, PLR, and, in a specific subcohort, N-terminal pro B-type natriuretic peptide (NT-proBNP), alongside length of stay (LOS) and 90-day readmission rates in CHF patients, irrespective of heart failure phenotype. A retrospective analysis of 427 CHF admissions (males = 57.84%) was conducted. Results: The mean age of the entire population was 68.48 ± 11.53 years. The average LOS was 8.33 ± 5.26 days, with a readmission rate of 73 visits (17.09%) for 56 patients. The NLR (3.79 ± 3.32) showed a low but positive correlation with the LOS (r = 0.222, *p* < 0.001). Conversely, the PLR (144.84 ± 83.08) did not demonstrate a significant association with the LOS. The NLR presented a low negative correlation for days until the next admission (r = −0.023, *p* = 0.048). In a prespecified subanalysis of 323 admissions, the NT-proBNP exhibited a low positive Pearson correlation with the NLR (r = 0.241, *p* < 0.001) and PLR (r = 0.151, *p* = 0.006). Conclusions: The impact of the NLR across heart failure phenotypes may suggest the role of systemic inflammation in understanding and managing CHF.

## 1. Introduction

Heart failure (HF) is a complex clinical syndrome characterized by the heart’s inability to pump blood sufficiently to fulfill the body’s needs. With an aging population and advances in cardiovascular therapy, the prevalence of heart failure is continuing to rise, making it a global public health issue. HF represents the final convergent point for a plethora of cardiovascular pathologies. Biomarkers play a critical role in the diagnosis, risk stratification, and management of heart failure [1,2].

Numerous inflammatory biomarkers are involved in myocardial tissue damage mediated by various oxidative stress cytokines across the entire range of heart failure phenotypes [3].

The pathophysiology of heart failure involves a broad spectrum of changes, including neurohormonal activation, molecular alterations, and altered hemodynamics. Inflammation is a critical component in the progression of heart failure. The neutrophil-to-lymphocyte ratio (NLR), a ratio of neutrophil to lymphocyte absolute numbers in the complete blood count, has emerged as a straightforward, cost-effective, and predictive biomarker in heart failure [4,5].

While the precise mechanisms by which NLR impacts heart failure outcomes are yet to be fully elucidated, they are generally believed to reflect the interplay between inflammatory activity and immune response, indicative of the body’s physiological stress reaction.

Neutrophils, which serve as the frontline defense in the immune system, are primarily associated with acute inflammation and tissue damage, whereas lymphocytes are involved in immunoregulation and healing processes [6,7]. The NLR thus represents a dynamic balance between these complementary roles: neutrophils’ pro-inflammatory effects and lymphocytes’ role in controlling chronic inflammation. This ratio offers valuable insights into a patient’s inflammatory state [8,9,10].

Over the past few decades, numerous studies have highlighted the potential relationship between inflammatory markers and the severity of heart failure [11,12].

Clinical research studies have investigated the role of the NLR in heart failure. Uthamalingam et al. demonstrated that the NLR could predict mortality in patients with acute heart failure independent of the left ventricular ejection fraction and a higher risk for 30-day acute decompensated HF-related readmission [8]. In a study published in 2015, Durmus et al. report that neutrophil-to-lymphocyte ratio (NLR) values are elevated in heart failure patients compared to matched controls and may serve as a predictor of mortality during follow-up [13]. In a comprehensive meta-analysis, Wang et al. concluded that elevated neutrophil-to-lymphocyte ratios are significant predictors of all-cause mortality and are correlated with renal lesions in patients with heart failure [14].

Another analysis performed by Di Rossa et al. on geriatric patients demonstrated the usefulness of the NLR in determining a high risk of mortality during hospital stays. Among other studied comorbidities, such as diabetes mellitus, metabolism and nutrition disorders, lung infections, and sepsis, patients with HF presented elevated values of NLR with a mean value of 10.06 [15]. The Rotterdam study, which assessed the NLR as a risk indicator of mortality in the general population, showed that it was a statistically significant factor in older patients [16].

Pang et al. suggest that a combined assessment of the NLR and red blood cell distribution width can be applied in the prompt detection of any developments, worsening prognosis, and gravity of HF in diabetic patients [17].

Zhu et al. investigated an inflammatory prognostic scoring method, demonstrating that the neutrophil-to-lymphocyte ratio (NLR) maintained its independence as a predictor of all-cause mortality in patients with acute heart failure [18]. Additionally, a meta-analysis by Ang et al. revealed that NLR values during index hospitalization were significantly higher in patients who experienced in-hospital mortality compared to survivors [19].

In a comprehensive review, Delcea et al. discussed the relationship between the NLR and heart failure and pointed out that the NLR is associated with the severity of heart failure, age, and comorbidity burden [20]. Higher NLR values were associated with in-hospital and short-term mortality, but they were not consistently an independent predictor of this outcome.

Zahorec et al. contributed an original paper based on several clinical trials, proposing an NLR meter exposing the normal NLR cut-off values [21]. They stated that, in adults, the normal range for NLR is considered to be from 1 to 2, and pathological values are below 0.7 and higher than 3.0.

Bhat et al. reported that a reduction in the NLR was associated with a shorter length of stay (LOS) following left ventricular assist device (LVAD) implantation [22].

Zhang et al. analyzed data from 26,021 patients included in the National Health and Nutrition Examination Survey (NHANES) between 2009 and 2018, finding that the NLR was strongly correlated with the risk of heart failure when evaluated using a multi-biomarker model. [23].

The NLR has been identified as a predictive marker in patients infected with COVID-19, and elevated values have been shown to influence clinical outcomes in individuals with bacterial pneumonia and malignancies [24,25,26]. Additionally, an increased NLR has been linked to higher risks of both all-cause and cardiovascular mortality in hypertensive patients [27].

Platelets are considered to be a bridge between inflammatory status, thrombosis, and hemostasis [28].

The platelet-to-lymphocyte ratio (PLR) is a ratio between the platelet’s absolute number and the lymphocyte’s absolute number and has been studied for its potential role in the prognostic assessment of patients with chronic heart failure (CHF) with reduced ejection fraction [29]. Thus, it is considered to be an indicator of both inflammation and thrombotic activity. Platelets and lymphocytes are actively involved in scenarios with heart failure and systemic inflammation, leading to the consideration of the PLR as a potential marker of disease severity.

An elevated NLR is significantly associated with an increased risk of all-cause mortality among community-dwelling adults with heart failure, underscoring its prognostic significance as an independent marker of inflammation-driven adverse outcomes in a study performed by Wu et al. [30]. Unlike the NLR, the PLR does not show a significant association with all-cause mortality in heart failure patients, suggesting limited utility as an independent prognostic biomarker in this population. In rheumatoid arthritis, the NLR and PLR can distinguish patients with or without active disease [31]. Cheng et al. identified the NLR and PLR as associated with the severity of disease in HF with reduced ejection fraction (HFrEF) and survivability on a par with cancer patients, while the PLR expressed higher risks than the neoplasia cohort [32].

While most published studies have concentrated on 30-day mortality as associated with these two biomarkers, the aim of this study was to evaluate the prognostic significance of the NLR and PLR in predicting length of stay, stratified by heart failure phenotypes. Additionally, it assessed total readmission rates within the first 30 days (early rehospitalization) post-discharge, as well as readmissions between 31 and 90 days, in patients with CHF.

In a subanalysis of our cohort, in which the N-terminal pro B-type natriuretic peptide (NT-proBNP) values were available, we hypothesized that the NT-proBNP, NLR, and PLR values could individually predict the LOS.

## 2. Materials and Methods

The primary dataset consisted of retrospective data from 1965 admissions to the Department of Internal Medicine II—Cardiology, County Emergency Clinical Hospital Targu Mures, Romania, from 1 January 2022 to 31 December 2023.

The inclusion criteria encompassed all medical records with a primary diagnosis of CHF, as defined by the International Classification of Diseases, 10th Revision (ICD-10), codes I50.0, I50.1, I50.9, and I51.7, and an LOS exceeding 48 h.

Conversely, the exclusion criteria included subjects with incomplete data, as well as those admitted with acute infectious conditions (viral, bacterial, or fungal infections), active inflammation, sepsis, active malignancies, autoimmune disorders, primary and viral hepatic diseases, hematologic malignancies, myelodysplastic syndromes, patients undergoing dialysis, those with acute or subacute venous thromboembolic diseases, and the absence of NT-proBNP values for heart failure with preserved ejection fraction (HFpEF) at diagnosis.

**Figure 1:**
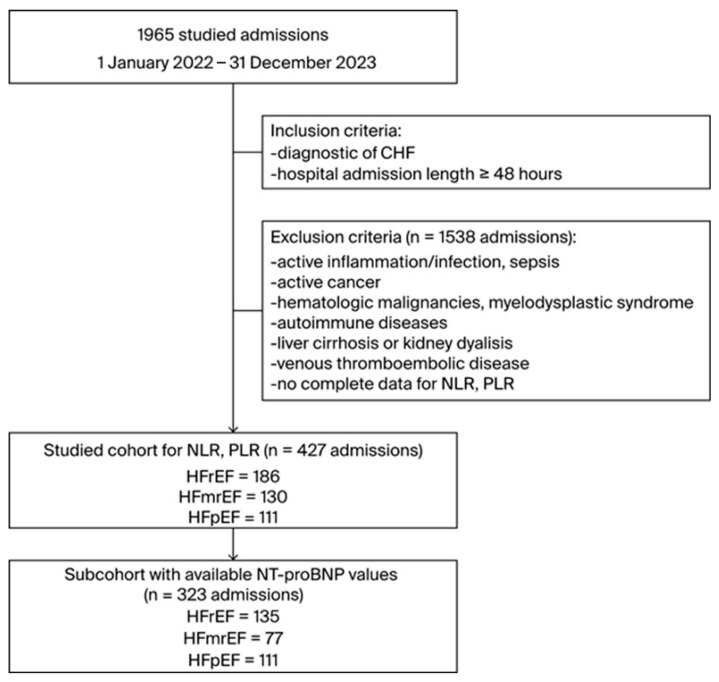
Flow diagram of study cohort selection. CHF, chronic heart failure; HFmrEF, heart failure with mildly reduced ejection fraction; HFpEF, heart failure with preserved ejection fraction; HFrEF, heart failure with reduced ejection fraction; n, number; NT-proBNP, N-terminal pro B-type natriuretic peptide; NLR, neutrophil-to-lymphocyte ratio; PLR, platelet-to-lymphocyte ratio.

We preserved the data for multiple admissions, and after applying initial inclusion and exclusion criteria, the sample was reduced to 355 patients, accounting for 427 admissions to be analyzed (see Figure 1). The mortality rate during admission was 3.04% (13 cases).

Furthermore, additional parameters considered for extended secondary analysis included demographic data, body mass index (BMI), primary vital signs such as blood pressure and heart rate, New York Heart Association (NYHA) functional class assessment, and a diagnosis of documented coronary artery disease, atrial fibrillation/flutter, valvular heart diseases ≥ moderate, hypertension, type II diabetes mellitus, and inflammatory biomarkers (erythrocyte sedimentation rate—ESR, fibrinogen). C-reactive protein was excluded from the analysis due to its inclusion of inhomogeneous data, which comprised both quantitative and qualitative representations in less than 15% of cases.

The BMI (Quetelet index) was calculated according to the classic formula: BMI (kg/m^2^) = weight (kg)/height (m)^2^.

New diagnoses of diabetes mellitus were confirmed by the 2021 and 2022 American Diabetes Association (ADA) criteria [33,34].

Blood samples for complete blood count (CBC) were collected via median cubital vein in the first two hours of admission and processed with a Sysmex XN-550 system (Sysmex Corporation, Kobe, Japan) in the on-site certified ISO 15189:2013 laboratory. The NLR and PLR were calculated once per admission from the CBC from the initial blood sample. Serum levels of NT-proBNP values were determined using a Nano-Checker™ 710 Reader (Nano-Ditech Corporation, Cranbury, NJ, USA).

Routine, guideline-aligned transthoracic echocardiography (TTE) evaluations of cardiac morphology and function were performed with a GE Vivid™ E9 System (GE Vingmed Ultrasound AS, Horten, Norway).

In line with the comprehensive framework delineated in the 2021 ESC Guidelines for the diagnosis and treatment of acute and chronic heart failure, the patient cohort recruited for this study was classified into three distinct groups reflective of varying degrees of cardiac dysfunction: HFrEF, HFmrEF, and HFpEF [1]. During the admission, pharmacological treatment was optimized to align with the standard of care based on current guidelines, utilizing all fundamental therapies in maximum tolerated dosages and taking into account the patient’s clinical status.

Three subgroups were defined for the NLR: group A (NLR < 3), group B (3 ≤ NLR ≤ 7), and group C (NLR > 7). Similarly, three subgroups were set for the PLR, group D (PLR < 150), group E (150 ≤ PLR ≤ 200), and group F (PLR > 200), to assess the impact of different cut-off values.

The minimum sample size was meticulously calculated to ensure robust statistical analyses to meet the desired statistical constraints. A population proportion of 30% was necessary to achieve a 95% confidence interval (CI) for the entire department for admissions over a period of two years, and a value of around 278 medical records was necessary to obtain a confidence level of 95% that the real value was within ±5% of the measured/surveyed value. The sample size was chosen to capture the variability within the population and allow for reliable inferences. We considered factors such as the confidence level, the desired precision level, and the study population’s inherent characteristics.

We gathered patient data using the Microsoft**^®^**Excel**^®^**2016 MSO version 16.0.4738.1000 for Windows (Microsoft Corporation, Redmond, WA, USA), and they were subsequently statistically analyzed using GraphPad Prism version 9.3.0 (436) for Windows, (GraphPad Software, Boston, MA, USA, www.graphpad.com, accessed on 18 March 2024). The normal distribution of the data was tested using the Shapiro–Wilk test. For intergroup comparison, the Kruskal–Wallis test was utilized. Categorical data are presented as frequencies (percentages), and continuous data are presented as the mean values ± standard deviation (SD). Statistical significance was considered when *p* ≤ 0.05. Correlations were determined using Spearman’s rank correlation coefficient for non-parametric variables, and parametric data were analyzed using Pearson correlation. A chi-squared test was used to compare categorical variables. Intergroup variation was analyzed using the ANOVA test for non-repeated measures, and after, the post hoc test was applied using the Dunn–Bonferroni test. Receiver operating characteristics (ROC) curve analysis was performed to determine the optimal cut-off values of NLR and PLR for early admission events. Kaplan–Meier curves were considered appropriate to display in figures the impact of the NLR and the PLR on rehospitalization events. To compare the survival distributions of the NLR and the PLR subgroups, the log-rank test (Mantel–Cox) was applied. To assess if there was a trend across these subgroups, the log-rank test for trends was administered.

## 3. Results

### 3.1. Cohort Characteristics

Data from 427 admissions diagnosed with CHF were analyzed (see Table 1). The mean age of the entire population was 68.48 ± 11.53 y.o. (IQR: 26–93 y.o.). There were 247 male patients (57.84%), with the majority of the cohort originating from urban areas (n = 298, 69.78%).

The majority of patients were categorized under NYHA functional classes II and III, with 11.47% classified as NYHA class IV. Atrial fibrillation or flutter was the most prevalent arrhythmia, observed in 41.15% of patients upon admission. Additionally, type 2 diabetes mellitus (T2DM) was present in 38.87% of the cohort.

### 3.2. Admission and Readmission

The overall length of stay was 8.33 ± 5.26 days, with noteworthy differences between groups. Patients presented longer hospital admission lengths according to the phenotype of ejection fraction; to be precise, HFrEF patients exhibited a longer LOS (*p* < 0.001). Extended LOS (>7 days) was found in 44.96% cases.

Furthermore, the study recorded a total of 56 patients with at least one readmission in the next 90 days from baseline, underscoring the ongoing healthcare burden posed by heart failure.

### 3.3. NLR and PLR among the Entire Cohort

Significant variance was observed in lymphocytes and platelets among the three HF phenotypes, but not for the neutrophils. For the analyzed ratios, the NLR exhibited differing distributions across the three HF phenotypes (*p* = 0.025, Kruskal–Wallis test), with an average ratio ≥ 3.79.

NLR and PLR did not exhibit correlations when compared with standard inflammatory biomarkers (ESR or fibrinogen).

The NLR presented a low, positive correlation with admission length (r = 0.22, *p* < 0.001). The PLR did not influence the duration of hospitalization (r = 0.08, *p* = 0.083), regardless of any phenotype of HF.

**Figure 2:**
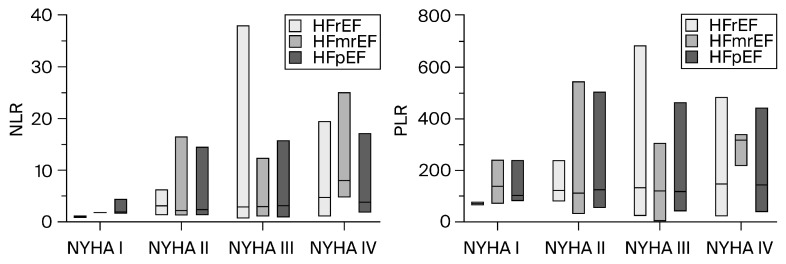
Distribution of NLR (**left panel**) and PLR (**right panel**) across NYHA functional classes. HFmrEF, heart failure with mildly reduced ejection fraction; HFpEF, heart failure with preserved ejection fraction; HFrEF, heart failure with reduced ejection fraction; NLR, neutrophil-to-lymphocyte ratio; NYHA, New York Heart Association; PLR, platelet-to-lymphocyte ratio. The horizontal line inside the floating bars refers to the median of the specified HF phenotype.

A significant difference in NLR values regarding NYHA functional classes (*p* < 0.001) was observed (see Figure 2). A post hoc test using the Dunn–Bonferroni test showed that the pairwise group comparisons of NYHA I with NYHA IV, NYHA II with NYHA III, NYHA II with NYHA IV, and NYHA III with NYHA IV were significantly different in pairs (*p* < 0.05).

While analyzing the relationship between the PLR and the functional NYHA classes, the medians varied significantly (*p* = 0.014, Kruskal–Wallis test; see Figure 2). The Dunn–Bonferroni test showed that NYHA II with NYHA IV had an adjusted *p*-value of less than 0.05, and thus, based on the available data, it was assumed that the two groups were significantly different from each other.

However, both the NLR and PLR presented a low, positive correlation with the NYHA functional class (r = 0.28, *p* < 0.001 for NLR; r = 0.15, *p* = 0.002 for PLR).

The NLR and PLR did not influence the extended LOS of the entire cohort (*p* > 0.05, Pearson correlation).

### 3.4. NLR and PLR Impact for the First Visit and Any Number of Readmissions < 90 Days

A comparison of patients with no readmissions in fewer than 90 days to those with one or more readmissions in the same time frame revealed no significant differences in NLR (*p* = 0.094) or PLR (*p* = 0.762). Additionally, gender did not significantly influence variations in NLR or PLR.

Kaplan–Meyer curves were considered appropriate to analyze the impact of NLR and PLR on rehospitalization events. Assessing the influence of NLR or PLR determined at baseline on the total number of readmissions/patient, no correlation of these two biomarkers was established.

**Figure 3:**
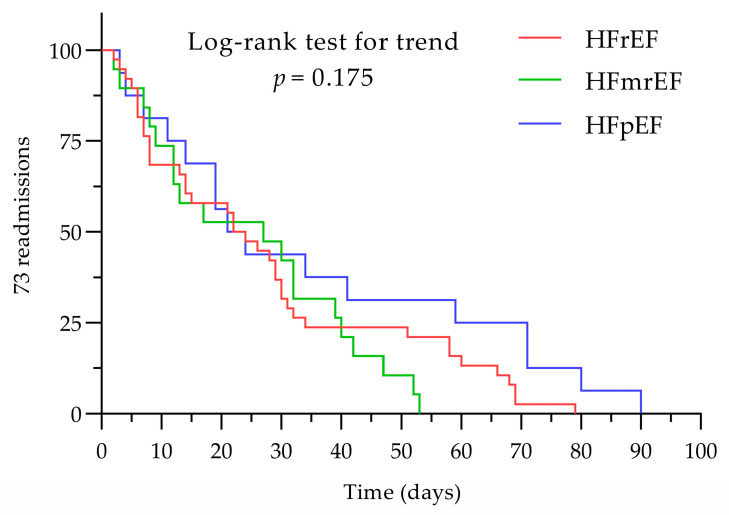
Readmission events within 90 days related to the HF phenotype. HFmrEF, heart failure with mildly reduced ejection fraction; HFpEF, heart failure with preserved ejection fraction; HFrEF, heart failure with reduced ejection fraction.

A log-rank test was calculated to test a possible difference regarding time event occurrence between HF phenotypes. For the present data, the log-rank test showed no difference between the groups regarding time distribution until the readmissions occurred (*p* = 0.175); results are presented in Figure 3.

### 3.5. Cut-Off Values for NLR and PLR as Markers of Prognosis for Earlier Readmission (<30 Days)

The influence of NLR for each readmission presented a low, negative correlation for predicting the number of days until the next admission occurred (r = −0.23, *p* = 0.048), and the influence of PLR was not significant (r = −0.23, *p* = 0.051).

**Figure 4:**
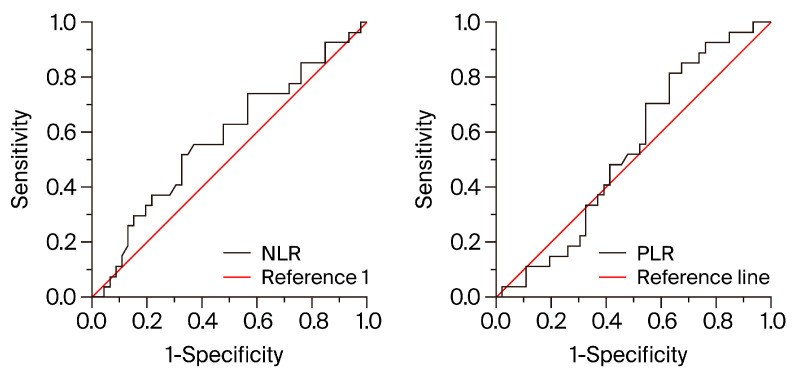
Receiver operating characteristic (ROC) curves for NLR (**left panel**) and PLR (**right panel**) in predicting early readmission. NLR, neutrophil-to-lymphocyte ratio; PLR, platelet-to-lymphocyte ratio.

Receiver operating characteristic (ROC) curves were constructed to assess the ability of the NLR and PLR to predict early hospitalization. The areas under the ROC curve for NLR and PLR were not significant: 0.578 (*p* = 0.278) and 0.534 (*p* = 0.218), respectively. The ROC curves are illustrated in Figure 4.

**Figure 5:**
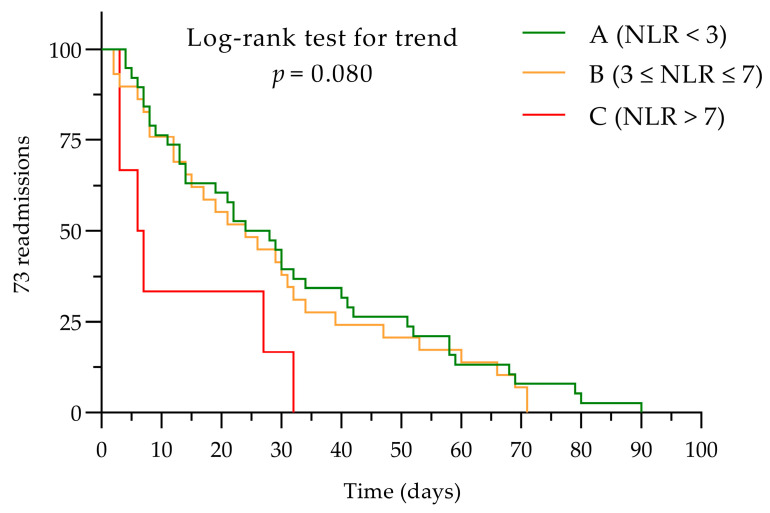
The NLR subgroup readmissions within 90 days after the first discharge. NLR, neutrophil-to-lymphocyte ratio.

In terms of readmission days curves, NLR subgroups presented significant distribution data (log-rank Mantel–Cox *p* = 0.046); however the trend did not have a significant impact according to log-rank test for trend (*p* = 0.080; see Figure 5).

**Figure 6:**
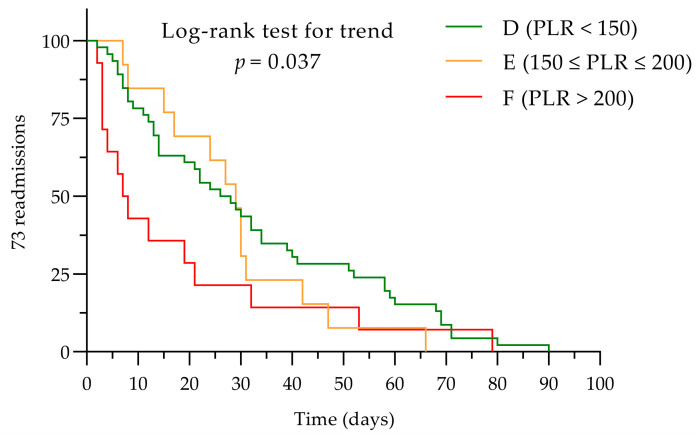
The PLR subgroups readmissions within 90 days. PLR, platelet-to-lymphocyte ratio.

Applying the log-rank Mantel–Cox test between subgroups D, E, and F, the data were not significantly distributed (*p* = 0.103), but higher PLR values were associated with earlier hospitalization events using the log-rank test for trend (*p* = −0.037; see Figure 6).

### 3.6. Reduced Cohort Size Data Comparison with the NT-proBNP

NT-proBNP is recognized globally as a biomarker correlated with admission length, mortality, and treatment initiation in HF patients. Due to the limited availability of NT-proBNP values, a subset of 323 files was selected from the initial cohort for a prespecified subanalysis using the values of NT-proBNP for reference. The data are depicted in Table 2.

In this subset, NT-proBNP exhibited a low positive Pearson correlation with NLR (r = 0.241, *p* < 0.001) and PLR (r = 0.151, *p* = 0.006). Significant variations in NT-proBNP levels were identified across all three heart failure phenotypes.

A strong direct correlation was observed between the NLR and PLR, as evidenced by a Pearson correlation coefficient of r = 0.723, *p* < 0.001.

Further analysis was conducted to identify correlations within specific HF phenotypes (see Table 3). Among these, the HFmrEF subjects diagnosed exhibited medium correlations between NT-proBNP—NLR and NT-proBNP—LOS.

### 3.7. Predictors for NLR and PLR in the NT-proBNP Subcohort

In the subcohort of 323 cases in which the values of NT-proBNP were available, a multilinear regression analysis using an ANOVA test identified age and NT-proBNP as consistent predictors of NLR (r^2^ = 0.074, *p* < 0.001). Regarding PLR, a significant statistical model was identified in only 4.3% of cases (r^2^ = 0.043, *p* = 0.007), with age and NT-proBNP as predictors.

In the two above-mentioned multilinear analyses, in a limited number of cases, age and NT-proBNP were repetitive factors that can influence NLR and PLR across multiple HF phenotypes.

## 4. Discussion

To the best of our knowledge, this is the first study that provides insights into the relationships between two biomarkers (NLR, PLR) and LOS in patients with CHF. NLR and PLR are increasingly recognized in clinical research, reflecting their potential in assessing the inflammatory state that significantly influences the progression and prognosis of heart failure [19,20,21,27,32]. This study aimed to elucidate the association between these two biomarkers and LOS in a moderate cohort sample size, highlighting the broader applicability of these simple, cost-effective measures in routine clinical practice.

In this analysis, the primary findings underscored a low, positive correlation between the NLR and the length of hospital stay in chronic HF patients. There were significant differences for NLR among HF phenotypes. NLR and PLR were correlated with the four functional NYHA classes. This suggests that, while NLR reflects an underlying non-specific systemic inflammatory response, its predictive utility may vary in accordance with HF phenotypes. This is consistent with the evidence suggesting that systemic inflammation is a fundamental pathophysiological mechanism in heart failure, contributing to myocardial remodeling, fibrosis and, ultimately, clinical decompensation leading to hospitalization [3]. However, as PLR is a biomarker linked to thrombotic activity, it was not influenced by HF phenotype, presumably underlining the contributions of other factors.

Chronic low-level inflammation and inadequate immune responses are linked to obesity [35,36]. Colluoglu et al. demonstrated that a NLR greater than 2.83 and an epicardial adipose tissue thickness exceeding 9.45 mm may serve as significant indicators for an increased risk of hospitalization in HFpEF patients [37]. In our research, 43.09% patients had a BMI > 29.99 kg/m^2^ at admission, mostly in the presence of congestion. Nonetheless, obesity can impact the outcome of several pathologic conditions and constitutes a major cardiovascular risk factor that must be taken into consideration. Another study aligns with these findings, strengthening the theory that inflammation biomarkers are correlated with levels of NLR in HFrEF and HFpEF and improved outcomes for patients presenting lower levels of NLR after 6 months in HFpEF [6].

A comprehensive systematic review and meta-analysis performed by Vakhshoori et al. identified only one study that examined the association between the extended LOS and the NLR, and it did not address the LOS in general as stratified by heart failure phenotypes [38]. Our study presents a novel approach by focusing on the correlation between the NLR, PLR and, for a specific subcohort, the NT-proBNP with LOS.

Delcea et al. examined a cohort of 1299 patients with heart failure, reporting an LOS greater than 7 days in 22.17% of cases [39]. Their findings identified the NLR as a significant predictor of prolonged LOS in HF patients, whereas the PLR did not show a significant association with admission duration across the entire cohort. Direct comparison between their study and ours is limited due to differences in inclusion criteria; their cohort included both acute and chronic HF patients, while our study focused specifically on those with chronic HF. Nevertheless, Delcea et al.’s research is among the few studies that investigate potential correlations between NLR, PLR, and LOS. In our study, an LOS longer than 7 days was identified in 44.96% of admissions. Our results state that the NLR and PLR did not show a significant correlation with extended length of stay across the entire patient cohort. This outcome is consistent with the existing literature, which has raised several questions regarding the reliability of PLR as an independent prognostic marker in heart failure [29,39].

Unlike the NLR, which consistently reflects inflammatory and stress responses, the PLR may be influenced by other variables, such as thrombotic states and hematological disorders, which can significantly vary among patients and might not directly correlate with the inflammatory burden [40,41].

Studies report a strong correlation for the NLR [5,8,22] but a scarce one for the PLR [29,40,42,43,44] concerning hospitalization and mortality events. However, within the studied time frame, our research encompassed only 73 readmissions derived from 56 patients.

We evaluated the relationship between NLR and PLR from prior hospitalizations and both the time to subsequent readmission and the total number of readmissions. No correlation was identified, regardless of heart failure phenotype. This finding may be due to the limited dataset available for this analysis. It is also well recognized that immune response declines with age, influenced by multiple factors. Our cohort was primarily composed of elderly patients (mean age: 68.48 years), overweight individuals (mean BMI: 29.78 kg/m^2^, affected by congestion), and a significant proportion with T2DM (38.87%). Other variables that could impact leukocyte or NT-proBNP levels, such as atrial fibrillation and/or chronic kidney disease, were not accounted for.

Moreover, the relationships observed between the NLR, NT-proBNP levels, and length of stay highlight the multifactorial nature of heart failure exacerbations requiring hospitalization. NT-proBNP is a well-established biomarker of cardiac stress and hemodynamic overload, and underlining its correlation with NLR in the actual study reinforces the interplay between hemodynamic instability and systemic inflammation. This interaction is pivotal in explaining the decompensation of heart failure that necessitates hospitalization, suggesting that a multi-marker model could enhance risk stratification and management strategies in CHF patients.

In our study, better correlations were obtained for HFmrEF and NLR, NT-proBNP levels, and LOS compared to the other two heart failure phenotypes. However, these findings should be interpreted with caution, as multiple other variables—both minor and major—could influence the underlying pathophysiological mechanisms, including the number of comorbidities, prolonged LOS due to extended comorbidity assessments in HFpEF, severe clinical status in HFrEF patients, and pharmacological treatments.

NLR demonstrated stronger correlations and a more consistent distribution compared to PLR in relation to NT-proBNP levels. This may be attributed to NLR being a more robust indicator of inflammation, as it includes two inflammatory components, unlike PLR, which incorporates one thrombotic and one inflammatory marker. As previously discussed, inflammation plays a key role in contributing to cardiac stress, as reflected by NT-proBNP release.

The implications of these findings extend to the management of heart failure, where markers such as the NLR could potentially guide therapeutic strategies aimed at mitigating inflammation in addition to conventional heart failure treatments. Anti-inflammatory molecules have been explored as possible options in heart failure therapies, and identifying patients with elevated NLR could facilitate the more effective tailoring of the modern five pillars used as fundamental HF therapy: (i) diuretics, (ii) sodium-glucose cotransporter-2 inhibitor, (iii) angiotensin-convertin enzyme inhibitors/angiotensin receptor-neprilysin inhibitor/angiotensin, (iv) receptor blockers/mineralocorticoid receptor antagonist, and (v) beta-blockers. Major cardiovascular events are prone to being reduced by the normalization of the NLR after anti-cytokine therapy [10]. Moreover, integrating the NLR and PLR into predictive models for hospitalization could facilitate earlier interventions aimed at preventing hospital readmissions, a significant burden in heart failure management.

This study has several limitations. First, as a single-center study, the generalizability of the results may be constrained. Second, the readmission analysis was limited to a 90-day period, potentially missing clinically significant readmissions beyond this timeframe. A larger cohort would have enhanced the statistical power and robustness of the findings. Gathering and analyzing quantitative C-reactive protein, in conjunction with the NLR and PLR, pharmacological treatment, or ventilatory support could yield more precise and accurate conclusions. Moreover, a comprehensive assessment of significant comorbidities could provide deeper insights into the burden and impact of these pathologies linked to inflammation in the context of HF.

## 5. Conclusions

This study explores the utility of the NLR and PLR as biomarkers of systemic inflammation for predicting the LOS in patients with CHF. The analysis revealed that the NLR presented a low but positive correlation with the LOS, whereas the PLR did not demonstrate such a relationship. The values of the NLR and PLR did not appear to influence the number of hospital days or the frequency of readmissions within 90 days overall. The NLR and PLR were linked to the severity of CHF assessed using the NYHA functional classification.

Future research should prioritize longitudinal assessments and explore the potential for integrating inflammatory markers with clinical management strategies to improve outcomes in heart failure patients. Developing multi-marker prognostic models incorporating NLR may provide a personalized approach to patient stratification and management, ultimately enhancing care quality and alleviating the hospitalization burden in chronic heart failure.

## Figures and Tables

**Table 1:** Characteristics of the entire studied group.

Parameter	Entire Cohort	HFrEF	HFmrEF	HFpEF	Intergroup Variation (*p*-Value)
Admissions (n)	427	186	130	111	-
Male	247	126	76	45	-
Urban	298	134	93	71	-
Age (years, mean ± SD)	68.48 ± 11.53	66.48 ± 11.53	69.14 ± 11.97	70.97 ± 9.52	0.002
BMI (kg/m^2^, mean ± SD)	29.78 ± 6.15	29.43 ± 5.77	29.48 ± 6.22	30.7 ± 6.63	0.254
HR (bpm, mean ± SD)	81.06 ± 22.24	85.83 ± 23.61	79.77 ± 22.62	75.07 ± 17.48	<0.001
SBP (mmHg, mean ± SD)	131.25 ± 23.61	127.24 ± 24.17	131.65 ± 21.33	137.51 ± 23.98	<0.001
DBP (mmHg, mean ± SD)	78.63 ± 13.5	78.57 ± 12.28	77.58 ± 12.94	79.95 ± 15.89	0.596
NYHA functional class (n)	-				
I	11	1	3	7	-
II	123	37	44	42	-
III	244	110	78	56	-
IV	49	38	5	6	-
Coronary artery disease (n)	71	35	20	16	-
Atrial fibrillation/flutter (n)	180	75	53	52	-
Valvular heart disease ≥ moderate (n)	160	67	48	45	-
Hypertension (n)	346	138	113	95	-
T2DM (n)	166	67	49	50	-
ESR (mm/h, mean ± SD)	17.85 ± 17.06	14.44 ± 12.7	17.16 ± 17.88	23.09 ± 19.88	0.001
Fibrinogen (g/L, mean ± SD)	4.08 ± 1.07	4.01 ± 1.08	3.99 ± 1.1	4.33 ± 1.01	0.031
Neutrophils (×10^3^/µL, mean ± SD)	5.58 ± 2.33	5.78 ± 2.63	5.37 ± 2.07	5.47 ± 2.03	0.622
Lymphocytes (×10^3^/µL, mean ± SD)	1.90 ± 1.15	1.88 ± 1.42	1.90 ± 0.89	1.95 ± 0.88	0.035
Platelets (×10^3^/µL, mean ± SD)	224.34 ± 71.67	216.12 ± 71.86	221.75 ± 62.98	241.15 ± 78.43	0.011
NLR (mean ± SD)	3.79 ± 3.32	4.05 ± 3.71	3.59 ± 3.06	3.61 ± 2.91	0.025
PLR (mean ± SD)	144.84 ± 83.08	144.08 ± 79.27	138.13 ± 73.42	153.97 ± 98.52	0.711
Admission length (days, mean ± SD)	8.33 ± 5.26	9.15 ± 5.79	7.88 ± 5.30	7.5 ± 4.00	<0.001
Extended LOS (n, >7 days)	192	97	52	43	0.542
Number of readmissions ≤ 90 days (n = 73 *)	HF phenotypes at readmission (n = 56 unique patients)				-
	HFrEF	HFmrEF		HFpEF	-
1	22	9		12	-
2	6	5		0	-
3	0	0		0	-
4	1	0		1	-

BMI, body mass index; bpm, beats per minute; DBP, diastolic blood pressure; ESR, erythrocyte sedimentation rate; HF, heart failure; HFmrEF, heart failure with mildly reduced ejection fraction; HFpEF, heart failure with preserved ejection fraction; HFrEF, heart failure with reduced ejection fraction; HR, heart rate; LOS, length of hospital stay; n, number; NLR, neutrophil-to-lymphocyte ratio; NYHA, New York Heart Association; PLR, platelet-to-lymphocyte ratio; SBP, systolic blood pressure; SD, standard deviation; T2DM, type 2 diabetes mellitus. * In seven cases, the patient’s ejection fraction degraded/improved/recuperated compared to the first presentation in the studied time frame.

**Table 2:** Subset characteristics.

Parameters	Entire Cohort	HFrEF	HFmrEF	HFpEF	Intergroup Variation (*p*-Value)
Patients (n)	323	135	77	111	-
NT-proBNP (pg/mL, mean ± SD)	4695.80 ± 7561.20	7197.80 ± 8957.43	3023.26 ± 4302.50	2813.10 ± 6603.80	<0.001
LOS (days, mean ± SD)	8.28 ± 5.32	8.87 ± 5.78	8.40 ± 6.02	7.50 ± 4.00	0.21
NLR (mean ± SD)	3.89 ± 3.46	4.15 ± 4.16	3.84 ± 2.78	3.61 ± 2.91	0.13
PLR (mean ± SD)	149.62 ± 89.49	149.03 ± 85.65	144.39 ± 83.03	153.97 ± 98.52	0.82

HFmrEF, heart failure with mildly reduced ejection fraction; HFpEF, heart failure with preserved ejection fraction; HFrEF, heart failure with reduced ejection fraction; LOS, length of stay; n, number; NLR, neutrophil-to-lymphocyte ratio; NT-proBNP, N-terminal pro B-type natriuretic peptide; PLR, platelet-to-lymphocyte ratio; SD, standard deviation.

**Table 3:** Correlations for each heart failure phenotype (n = 323).

Parameters	NT-proBNP					
	HFrEF (n = 135)		HFmrEF (n = 77)		HFpEF (n = 111)	
	r *	*p*	r	*p*	r	*p*
LOS	0.19	0.03	0.56	<0.001	−0.01	0.88
NLR	0.21	0.017	0.4	<0.001	0.24	0.013
PLR	0.17	0.048	0.25	0.028	0.13	0.182

HFrEF, heart failure with reduced ejection fraction; HFmrEF, heart failure with mildly reduced ejection fraction; HFpEF, heart failure with preserved ejection fraction; LOS, length of stay; NLR, neutrophil-to-lymphocyte ratio; NT-proBNP, N-terminal pro B-type natriuretic peptide; PLR, platelet-to-lymphocyte ratio; * r value derived from the Pearson correlation.

## Data Availability

Supplementary information can be obtained from the corresponding author according to the local and national regulations.

## References

[1] McDonagh T.A., Metra M., Adamo M., Gardner R.S., Baumbach A., Böhm M., Burri H., Butler J., Čelutkienė J., Chioncel O. (2021). 2021 ESC Guidelines for the diagnosis and treatment of acute and chronic heart failure. Eur. Heart J..

[2] Bui A.L., Horwich T.B., Fonarow G.C. (2011). Epidemiology and risk profile of heart failure. Nat. Rev. Cardiol..

[3] Mann D.L. (2015). Innate immunity and the failing heart: The cytokine hypothesis revisited. Circ. Res..

[4] Balta S., Celik T., Mikhailidis D.P., Ozturk C., Demirkol S., Aparci M., Iyisoy A. (2016). The Relation Between Atherosclerosis and the Neutrophil-Lymphocyte Ratio. Clin. Appl. Thromb. Hemost..

[5] Bhat T., Teli S., Rijal J., Bhat H., Raza M., Khoueiry G., Meghani M., Akhtar M., Costantino T. (2013). Neutrophil to lymphocyte ratio and cardiovascular diseases: A review. Expert Rev. Cardiovasc. Ther..

[6] Curran F.M., Bhalraam U., Mohan M., Singh J.S., Anker S.D., Dickstein K., Doney A.S., Filippatos G., George J., Metra M. (2021). Neutrophil-to-lymphocyte ratio and outcomes in patients with new-onset or worsening heart failure with reduced and preserved ejection fraction. ESC Heart Fail..

[7] Mayadas T.N., Cullere X., Lowell C.A. (2014). The multifaceted functions of neutrophils. Annu. Rev. Pathol..

[8] Uthamalingam S., Patvardhan E.A., Subramanian S., Ahmed W., Martin W., Daley M., Capodilupo R. (2011). Utility of the neutrophil to lymphocyte ratio in predicting long-term outcomes in acute decompensated heart failure. Am. J. Cardiol..

[9] Horne B.D., Anderson J.L., John J.M., Weaver A., Bair T.L., Jensen K.R., Renlund D.G., Muhlestein J.B., Intermountain Heart Collaborative Study Group (2005). Which white blood cell subtypes predict increased cardiovascular risk?. J. Am. Coll. Cardiol..

[10] García-Escobar A., Vera-Vera S., Tébar-Márquez D., Rivero-Santana B., Jurado-Román A., Jiménez-Valero S., Galeote G., Cabrera J.Á., Moreno R. (2023). Neutrophil-to-lymphocyte ratio an inflammatory biomarker, and prognostic marker in heart failure, cardiovascular disease and chronic inflammatory diseases: New insights for a potential predictor of anti-cytokine therapy responsiveness. Microvasc. Res..

[11] Mann D.L. (2002). Inflammatory mediators and the failing heart: Past, present, and the foreseeable future. Circ. Res..

[12] Van Linthout S., Tschöpe C. (2017). Inflammation—Cause or Consequence of Heart Failure or Both?. Curr. Heart Fail. Rep..

[13] Durmus E., Kivrak T., Gerin F., Sunbul M., Sari I., Erdogan O. (2015). Neutrophil-to-Lymphocyte Ratio and Platelet-to-Lymphocyte Ratio are Predictors of Heart Failure. Arq. Bras. Cardiol..

[14] Wang X., Fan X., Ji S., Ma A., Wang T. (2018). Prognostic value of neutrophil to lymphocyte ratio in heart failure patients. Clin. Chim. Acta.

[15] Di Rosa M., Sabbatinelli J., Soraci L., Corsonello A., Bonfigli A.R., Cherubini A., Sarzani R., Antonicelli R., Pelliccioni G., Galeazzi R. (2023). Neutrophil-to-lymphocyte ratio (NLR) predicts mortality in hospitalized geriatric patients independent of the admission diagnosis: A multicenter prospective cohort study. J. Transl. Med..

[16] Fest J., Ruiter T.R., Groot Koerkamp B., Rizopoulos D., Ikram M.A., van Eijck C.H.J., Stricker B.H. (2019). The neutrophil-to-lymphocyte ratio is associated with mortality in the general population: The Rotterdam Study. Eur. J. Epidemiol..

[17] Pang J., Qian L.Y., Lv P., Che X.R. (2024). Application of neutrophil-lymphocyte ratio and red blood cell distribution width in diabetes mellitus complicated with heart failure. World J. Diabetes.

[18] Zhu X., Cheang I., Xu F., Gao R., Liao S., Yao W., Zhou Y., Zhang H., Li X. (2022). Long-term prognostic value of inflammatory biomarkers for patients with acute heart failure: Construction of an inflammatory prognostic scoring system. Front. Immunol..

[19] Ang S.P., Chia J.E., Jaiswal V., Hanif M., Iglesias J. (2024). Prognostic Value of Neutrophil-to-Lymphocyte Ratio in Patients with Acute Decompensated Heart Failure: A Meta-Analysis. J. Clin. Med..

[20] Delcea C., Buzea C.A., Dan G.A. (2019). The neutrophil to lymphocyte ratio in heart failure: A comprehensive review. Rom. J. Intern. Med..

[21] Zahorec R. (2021). Neutrophil-to-lymphocyte ratio, past, present and future perspectives. Bratisl. Lek. Listy.

[22] Bhat G., Yost G.L., Ibrahim K., Pappas P., Tatooles A. (2018). Risk stratification with longitudinal neutrophil to lymphocyte ratio assessment after left ventricular assist device implantation. Int. J. Artif. Organs.

[23] Zhang Y., Feng L., Zhu Z., He Y., Li X. (2024). Association between blood inflammatory indices and heart failure: A cross-sectional study of NHANES 2009–2018. Acta Cardiol..

[24] Liu J., Yao L., Xiang P., Lin P., Xiong H., Li C., Zhang M., Tan J., Xu Y., Song R. (2020). Neutrophil-to-Lymphocyte Ratio Predicts Critical Illness Patients with 2019 Coronavirus Disease in the Early Stage. J. Transl. Med..

[25] Ge Y.L., Zhang H.F., Zhang Q., Zhu X.Y., Liu C.H., Wang N., Zhang J.B., Chen H., Chen Y., Li W.Q. (2019). Neutrophil-to-Lymphocyte Ratio in Adult Community-Acquired Pneumonia Patients Correlates with Unfavorable Clinical Outcomes. Clin. Lab..

[26] Cupp M.A., Margarita C., Tzoulaki I., Aune D., Evangelou E., Berlanga-Taylor A.J. (2020). Neutrophil to Lymphocyte Ratio and Cancer Prognosis: An Umbrella Review of Systematic Reviews and Meta-Analyses of Observational Studies. BMC Med..

[27] Zhang X., Wei R., Wang X., Zhang W., Li M., Ni T., Weng W., Li Q. (2024). The neutrophil-to-lymphocyte ratio is associated with all-cause and cardiovascular mortality among individuals with hypertension. Cardiovasc. Diabetol..

[28] Franco A.T., Corken A., Ware J. (2015). Platelets at the interface of thrombosis, inflammation, and cancer. Blood.

[29] Mongirdienė A., Laukaitienė J., Skipskis V., Kuršvietienė L., Liobikas J. (2021). Platelet Activity and Its Correlation with Inflammation and Cell Count Readings in Chronic Heart Failure Patients with Reduced Ejection Fraction. Medicina.

[30] Wu C.C., Wu C.H., Lee C.H., Cheng C.I. (2023). Association between neutrophil percentage-to-albumin ratio (NPAR), neutrophil-to-lymphocyte ratio (NLR), platelet-to-lymphocyte ratio (PLR) and long-term mortality in community-dwelling adults with heart failure: Evidence from US NHANES 2005–2016. BMC Cardiovasc. Disord..

[31] Zinellu A., Mangoni A.A. (2023). Neutrophil-to-lymphocyte and platelet-to-lymphocyte ratio and disease activity in rheumatoid arthritis: A systematic review and meta-analysis. Eur. J. Clin. Investig..

[32] Arfsten H., Cho A., Prausmüller S., Spinka G., Novak J., Goliasch G., Bartko P.E., Raderer M., Gisslinger H., Kornek G. (2021). Inflammation-Based Scores as a Common Tool for Prognostic Assessment in Heart Failure or Cancer. Front. Cardiovasc. Med..

[33] American Diabetes Association (2021). Standards of Medical Care in Diabetes-2021 Abridged for Primary Care Providers. Clin. Diabetes.

[34] American Diabetes Association (2022). Standards of Medical Care in Diabetes-2022 Abridged for Primary Care Providers. Clin. Diabetes.

[35] Ilich J.Z., Kelly O.J., Kim Y., Spicer M.T. (2014). Low-grade chronic inflammation perpetuated by modern diet as a promoter of obesity and osteoporosis. Arh. Hig. Rada Toksikol..

[36] de Heredia F.P., Gómez-Martínez S., Marcos A. (2012). Obesity, inflammation and the immune system. Proc. Nutr. Soc..

[37] Colluoglu T., Akın Y. (2023). The Value of Neutrophil-to-Lymphocyte Ratio and Epicardial Adipose Tissue Thickness in Heart Failure with Preserved Ejection Fraction. Cureus.

[38] Vakhshoori M., Nemati S., Sabouhi S., Yavari B., Shakarami M., Bondariyan N., Emami S.A., Shafie D. (2023). Neutrophil to lymphocyte ratio (NLR) prognostic effects on heart failure; a systematic review and meta-analysis. BMC Cardiovasc. Disord..

[39] Delcea C., Buzea C.A., Vijan A., Draghici A., Stoichitoiu L.E., Dan G.A. (2021). Comparative role of hematological indices for the assessment of in-hospital outcome of heart failure patients. Scand. Cardiovasc. J..

[40] Kim M.J., Kwon S.S., Ji Y.S., Lee M.Y., Kim K.H., Lee N., Park S.K., Won J.H., Yoon S.Y. (2023). Neutrophil-to-lymphocyte ratio and platelet-to-lymphocyte ratio as new possible minor criteria for diagnosis of polycythemia vera. Int. J. Lab. Hematol..

[41] Ren L., Xu J., Li J., Xu T., Yang Y., Wang W., Ren Y., Gu S., Chen C., Wei Z. (2023). A prognostic model incorporating inflammatory cells and cytokines for newly diagnosed multiple myeloma patients. Clin. Exp. Med..

[42] Delcea C., Buzea C.A., Vîjan A.E., Bădilă E., Dan G.A. (2023). The platelet to lymphocyte ratio in heart failure: A comprehensive review. Rom. J. Intern. Med..

[43] Turcato G., Sanchis-Gomar F., Cervellin G., Zorzi E., Sivero V., Salvagno G.L., Tenci A., Lippi G. (2019). Evaluation of Neutrophil-lymphocyte and Platelet-lymphocyte Ratios as Predictors of 30-day Mortality in Patients Hospitalized for an Episode of Acute Decompensated Heart Failure. J. Med. Biochem..

[44] Liu Z., Zhou X. (2022). A nomogram based on systemic inflammation markers can predict adverse outcomes in patients with heart failure. Nan Fang Yi Ke Da Xue Xue Bao.

